# Multimodal Local Resonators for Low-Frequency Amelioration of Acoustic Black Holes

**DOI:** 10.3390/ma16134579

**Published:** 2023-06-25

**Authors:** Jing Zhao, Zhixin Ma, Yiyang Hu, Jiacheng Zeng, Yuxin Xu, Jie Deng, Nansha Gao

**Affiliations:** 1 Key Laboratory of Ocean Acoustic and Sensing, School of Marine Science and Technology, Northwestern Polytechnical University, Xi’an 710072, China; 2 Key Laboratory of Unmanned Underwater Vehicle, School of Marine Science and Technology, Northwestern Polytechnical University, Xi’an 710072, China

**Keywords:** acoustic black holes, multimodal resonators, acoustic metamaterials, vibration reduction, low frequency

## Abstract

Acoustic black holes (ABHs) are effective at suppressing vibrations at high frequencies, but their performance at low frequencies is limited. This paper aims to improve the low-frequency performance of ABH plates through the design of a metamaterial acoustic black hole (MMABH) plate. The MMABH plate consists of a double-layer ABH plate with a set of periodic local resonators installed between the layers. The resonators are tuned to the low-frequency peak points of the ABH plate, which are identified using finite element analysis. To dissipate vibration energy, the beams of the resonators are covered with damping layers. A modal analysis of the MMABH plate is performed, confirming its damping effect over a wide frequency band, especially at low frequencies.

## 1. Introduction

The acoustic black hole (ABH) is an acoustic analogy of the concept of a black hole in astrophysics within the realm of mechanics. In 1946, Pekeris [1] discovered that waves in acoustics behave like black holes when sound waves propagate in an inhomogeneous stratified fluid. As the depth of the medium increases, the velocity of the waves decreases to zero, causing them to no longer reflect. The sound waves are unable to escape the boundary of the open area. In 1988, Mironov [2] discovered that the propagation of bending waves in wedge structures is also similar to the behavior of black holes; when a thin plate is tailored according to the power law, it results in a zero thickness and zero-reflection condition. In 1989, Krylov [3] studied the one-dimensional wedge-shaped beam structure and found that changing the geometric shape parameters of the wedge structure controls the propagation of bending waves. The concept of ABHs was clearly introduced as a change in geometric parameters or material properties. In this case, the bending wave velocity gradually decreases in the ABH region, forming a structure that has a convergence effect on bending waves.

In recent years, there have been numerous applications based on ABH, and research in this field has expanded into four areas: vibration reduction, wave regulation, noise radiation, and energy recovery. Lagny et al. [4] studied the nonlinear behavior of the ABH center and evaluated the efficiency of the notch filter by examining its reflection coefficient for vibration reduction. Zhao [5] proposed a composite structure that combines ABHs and sandwich plates for vibration reduction. Local resonant acoustic metamaterials with periodic resonators have been widely studied for vibration reduction [6,7,8], sound suppression [9,10,11], and wave manipulation [12,13]. In terms of wave regulation, a numerical study [14] demonstrated that the lensing and focusing effects were experimentally proven by using a line source to excite Lamb waves in a flat plate embedded in a two-dimensional circular ABH [15]. The influence of two-dimensional circular ABHs on the high-frequency bending wave propagation was revealed based on the earlier research of Yan et al. [16] and Lomonosov et al. [17] using geometric acoustic theory. With regard to noise radiation, Du et al. [18], Ji et al. [19], and Feurtado and Conlon [20] tested the sound insulation performance of two-dimensional ABH sheets using an acoustic conduit and an acoustic reverberation chamber. Li and Ding [21] found that acoustic radiation could be reduced by strengthening the interaction between bending waves and ABH structures. In terms of energy harvesting, Ji et al. [22] proposed and studied an energy collection structure with ABH characteristics. From the perspective of practical applications, ABH structures are expected to be used in industrial equipment preparation design, architectural acoustics, and functional composite design.

The ABH plate has several advantages over other methods in terms of vibration reduction and sound radiation, but also has some limitations. One of the limitations is that it only operates above the cut-on frequency $f_{\mathrm{cut}-\mathrm{on}}$ [23]. This means that the plate can only absorb vibrations above a certain frequency and not below this frequency. When the wavelength of the impact wave is larger than the diameter of the ABH plate, the impact wave “crosses” the ABH plate, and the ABH plate fails to work. This flaw in the ABH plate limits its effectiveness in attenuating low-frequency noise, which is highly penetrating and challenging to attenuate using conventional materials that are limited by the law of mass action in the provision of adequate sound insulation. It is, therefore, crucial to broaden the low-frequency bandwidth of the structure to attenuate low-frequency noise. One expected result is that the impact waves acting outside the ABH plate would concentrate in the center of the plate and dissipate energy by damping [24].

Few methods have been proposed to improve the low-frequency performance of ABH to date. In 2014, Feurtado investigated the reflection of flexural waves via ABHs when introduced to uniform beams without anechoic terminations through numerical simulations [25]. Foucaud further extended the use of ABH to artificial cochleas [26]. In addition, Conlon and Feurtado investigated the admittance response of the two-dimensional ABHs at low, moderate, and high frequencies using experimental and finite element approaches [27,28]. Since then, more researchers have studied ABHs. In 2017, Tang and Cheng proposed the beam structure of a one-dimensional double-leaf ABH and confirmed that ABH can achieve significant vibration energy attenuation in an ultra-wide frequency range, including low frequencies [29]. Deng solved the problem of how to alleviate the ABH vibrations of complex cylindrical shell structures [30], and Sheng used multiple acoustic black hole dynamic vibration absorbers (ABH-DVA) to reduce the transverse vibration of the beam structure [31]. Moreover, many studies have aimed to obtain a better damping effect by adjusting the ABH structure and the configuration of damping layers. For example, a PZT patch was pasted on the cantilever beam to improve its dynamic effect [32], and a shunt damper with a blocking circuit was pasted on one side of the central area of the ABH [33]. Additionally, a combination of ABH and techniques with different physical application backgrounds may be effective at low frequencies. For example, low frequencies can be converted to high frequencies and transmitted to the ABH, which can be used more efficiently. In that case, nonlinear, geometric, material, and contact methods can be used to transfer low-frequency energy to a more efficient high-frequency range to improve the passive damping ability of the ABH [34,35,36,37,38,39,40]. Negative stiffness elements also exhibit a good low-frequency performance. Researchers have combined ABH beams with negative stiffness elements (NS-ABH beams) to improve the low-frequency performance of ABH [41]. Moreover, metamaterials have excellent properties. In 2021, the wave attenuation and energy transfer mechanism of metamaterials with a negative effective mass density were discovered, providing a similar research method [42]. Deng proposed the design of a metamaterial ABH plate and studied the characteristics of its acoustic radiation [43,44]. Although acoustic metamaterials have attracted much attention and research, achieving a broadband frequency performance with locally resonant metamaterials remains challenging.

This paper presents two main contributions. Firstly, it proposes a double-layered ABH plate consisting of two identical ABH plates with circular indentations, connected by supporting bars. The design enhances the structure’s rigidity and allows for multimodal local resonators to be mounted on the supporting bars. Secondly, the paper presents a real multimodal resonator design that can suppress multiple low-frequency peaks. Unlike previous theoretical and single-modal designs, this resonator is based on a cantilever beam-type local oscillator, with four oscillators mounted on a single supporting bar to achieve a multimodal design. The resonators are tuned to form band gaps that suppress low-frequency vibration peaks in the double-layer ABH plate, while their beams are damped to reduce other vibration levels in the lower frequency bands. The interaction of the multimodal local resonator with the double-layer ABH plate is investigated in this work. Importantly, the mass of the ABH indentation that is removed from the uniform plate is recycled as the mass of the resonators, ensuring that the MMABH design does not increase the weight of the original uniform plate.

The organization of the paper is as follows. Section 2 explains the double-layer ABH plate’s configuration, including supporting bars. The design process for the MMABH plate is then described, which entails attaching the multimode local resonator to the supporting bar of the double-layer ABH plate. After completing the design of the MMABH plate, it is simulated and tested in Section 3. The results of the modal analysis performed on the MMABH plate are presented, and the MMABH plate’s ability to reduce broad frequency vibrations caused by external forces is analyzed. Finally, the effects of beam damping and multimodal local resonators are investigated.

## 2. Design of the Metamaterial ABH (MMABH)

In this section, the MMABH structure is described. At the outset, a high-stiffness double-layer ABH plate was proposed and its mean square velocity (MSV) was analysed when subjected to external forces. Peaks in the lower-frequency bands were observed, based on which multimodal local resonators were designed and tuned to exhibit band gaps at these peaks. In addition, an overlay was applied to dampen the resonators, significantly reducing the peaks at the remaining lower-order eigenfrequencies. Finally, the design of the MMABH plate was completed by integrating the multimodal local resonators with the supporting bars of the double-layer ABH plate, resulting in broadband damping that covers the entire frequency range, especially at low frequencies. This approach resulted in a highly effective solution for mitigating vibration-induced noise in ABH plates.

### 2.1. Double-Layer ABH Plate

**Figure 1:**
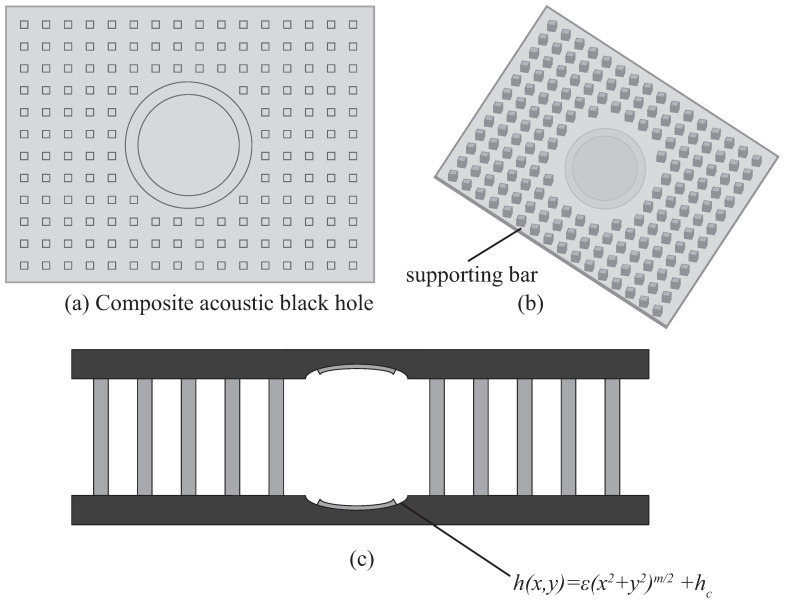
(**a**) Vertical view of the ABH. (**b**) Configuration of supporting bars on the bottom surface of the ABH plate. (**c**) ABH plate profile.

Geometric and material parameters for a single ABH plate configuration are provided in Table 1. The plate is uniform with a circular ABH indentation placed at its centre, and a circular thin viscoelastic layer of thickness $h_{v}$ and radius $r_{v}$ is attached at the ABH centre to dissipate energy. As shown in Figure 1, the ABH indentation has a power-law thickness $h\left(x,y\right)=\varepsilon {\left(x^{2}+y^{2}\right)}^{m/2}+h_{c}$. However, a single-layer ABH plate has low stiffness [45] and is not suitable for practical applications. Thus, a double-layer ABH plate was designed to solve this problem. The double-ABH plate consists of two identical ABH plates connected by supporting bars. The supporting bars were evenly distributed on the part of the plate that maintains the same thickness, dividing $L_{x}$ and $L_{y}$ into 16 and 12 equal sections, resulting in 160 supporting bars. To prevent the internal centre of ABH from contact with other parts, which can cause a loss of energy-gathering effect [45], the side of the ABH thickness variation is placed downwards to form a hollow drum. In this way, the two hollows are opposite each other, as shown in Figure 1c.

Finally, the purpose of designing this structure is presented. The double-layer ABH plate designed in this study has a black hole radius of 0.15 mm, which provides a suitable shear range and better load-bearing performance. Moreover, the supporting bars offer a convenient location for mounting the resonators.

**Figure 2:**
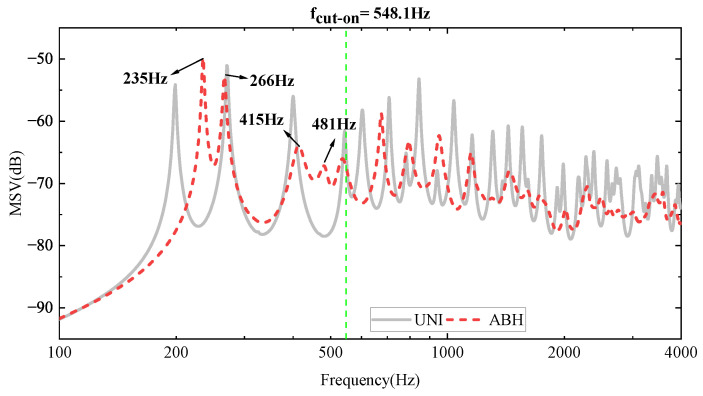
Mean square velocity (MSV) comparison between the ABH plate and the uniform (UNI) one.

After introducing the structure of the double-layer ABH plate, it was modelled using the finite element software COMSOL Multiphysics 6.0. The simulation was kept simple by setting simple support boundary conditions around the boundaries of both upper and lower plates and applying external forces to observe the structure’s response. The force was assumed to have a constant amplitude of 2 N for all frequencies, directed vertically towards the plate, and located at (0.3, 0, 0.0275) m. Its mean squared speed was then calculated (MSV, as shown in Figure 2).

(1) $$\mathrm{MSV}\left(\mathrm{dB}\right)=10\log\left[\int_{-L_{x}/2}^{L_{x}/2}\int_{-L_{y}/2}^{L_{y}/2}\omega^{2}w^{2}\left(x,y\right)dxdy/\left(L_{x}L_{y}\right)\right]$$

The calculated data from Table 1 enable the determination of the frequency at which the active black hole (ABH) begins to take effect. Specifically, the cut-on frequency $f_{\mathrm{cut}-\mathrm{on}}$ can be expressed as:

(2) $$f_{\mathrm{cut}-\mathrm{on}}=\frac{\pi h_{\mathrm{uni}}}{4r_{\mathrm{abh}}^{2}}\sqrt{\frac{E_{p}}{3\rho_{p}\left(1-v_{p}^{2}\right)}}=548.1\;\mathrm{Hz}$$

Therefore, for frequencies above 548.1 Hz, the double-layer ABH will function, leading to a significant reduction in vibration, as observed from the graph. However, for lower frequencies, the double-layer ABH is completely inefficient and will even work in the opposite direction (see Figure 2), which is very obvious in the first resonant frequency of the double-layer ABH plate, $f_{\mathrm{low}1\;}=235\;\mathrm{Hz}$. To address this issue, multimodal local resonators were designed in three iterations and mounted on supporting bars. This strategy created bandgaps in the low-frequency range, thereby improving the low-frequency performance of the double-layer ABH plates.

### 2.2. Multimodal Local Resonators

**Figure 3:**
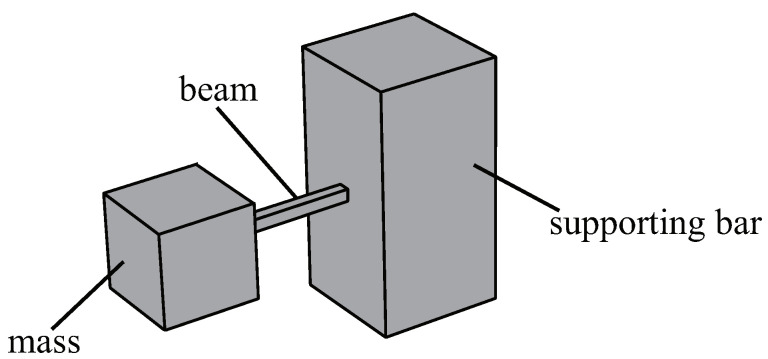
Geometry of one resonator connected to the supporting bar.

**Figure 4:**
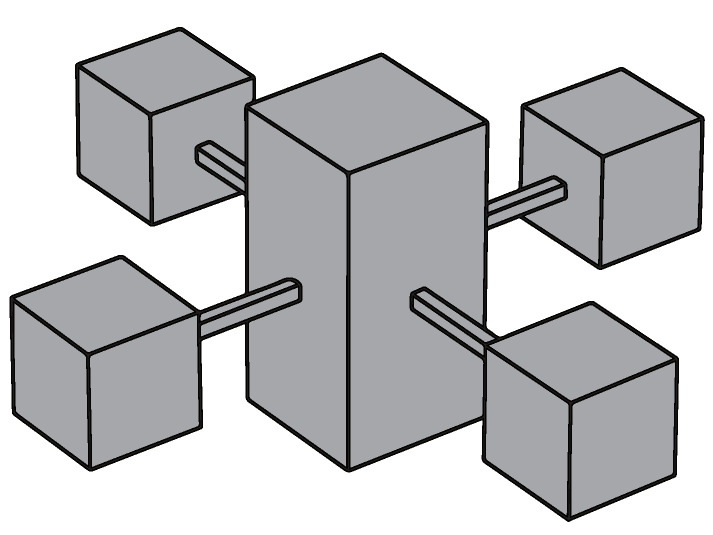
Multimodal local resonators without damping.

The periodic arrangement of small local resonators on the surface of an ABH plate structure serves the purpose of reducing the surface vibration velocity under the excitation of low-frequency vibration line spectra, enabling broad-spectrum vibration damping across the entire frequency range. Previous research has proposed a structure for periodically arranging spring oscillators on the back of the ABH to improve the performance of the ABH plate at low frequencies [43]. In this study, a structure is proposed that connects a multimodal local resonator to a double-layer ABH plate. A modified cantilever beam-type local field oscillator is designed to meet the demands for a low-frequency resonance and light weight (refer to Figure 3). As shown in Figure 2, there are two peaks to be reduced; therefore, the local resonator was designed as a multimodal local resonator that contains four oscillators (refer to Figure 4).

According to the geometrical and material parameters in Table 1, the mass extracted from the homogeneous plate is $M=2\rho_{p}V=2.2447\;\mathrm{kg}$, where *V* is the total volume removed when forming an ABH.

$$V=\int_{0}^{2\pi }d\varphi \int_{0}^{r_{\mathrm{abh}}}h_{\mathrm{uni}}rdr-\int_{0}^{2\pi }d\varphi \int_{0}^{r_{\mathrm{abh}}}\left(\varepsilon r^{m}+h_{c}\right)rdr=\pi r_{\mathrm{abh}}^{2}\left(h_{\mathrm{uni}}-h_{c}\right)-\frac{2\pi \varepsilon }{m+1}r_{\mathrm{abh}}^{m+2}$$

(3) $$=1.4389\times 10^{-4}\;m^{3}$$

Each supporting bar is equipped with four mass blocks, each with an assigned weight of approx Δm=M/160/4=0.0035kg. Assuming that each mass block is a cubic iron block, its dimensions are $a=\sqrt[{3}]{\frac{\Delta m}{\rho_{p}}}\approx 7.66\;\mathrm{mm}$. The mass of the removed ABH portion of the uniform plate is recycled as the mass of the resonators. As a result, the MMABH involves no increase in weight compared to the original homogeneous plate.

The elastic beam that connects the supporting bar to the mass block is made of the same material as the ABH plate. While keeping the parameters of the mass block unchanged, the length and cross-sectional area of the beam are adjusted to control its first-order eigenfrequency to be equal to the frequency point to be damped. The dimensions and eigenfrequencies of the three types of oscillators designed by parametric scanning are shown in Table 2.

**Figure 5:**
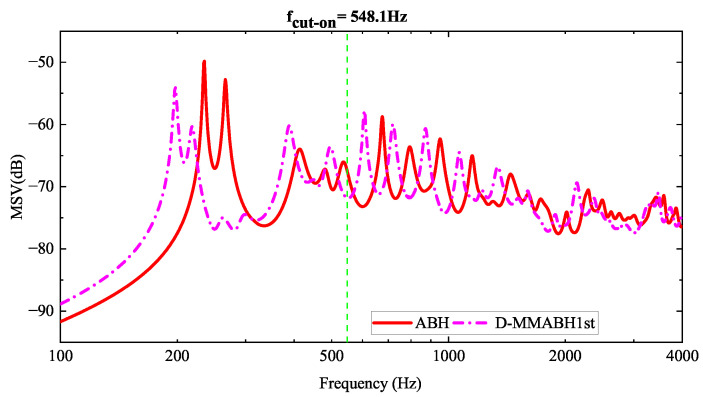
Mean square velocity (MSV) comparison between the ABH plate and the D-MMABH-1st plate.

From Figure 2, it is evident that the ABH plate exhibits a higher peak at the first resonant frequency $f_{\mathrm{low}1\;}=235\;\mathrm{Hz}$ compared to the uniform plate due to the reduced stiffness caused by the indentation of the ABH. Therefore, to attenuate the resonance peak at low frequencies, a local resonator is introduced, and three iterations are considered to tune the resonator. The designed multimodal local resonator comprises four oscillators that are tuned to three different frequencies. The two higher peak frequencies, 235.5 Hz and 265.99 Hz, were first considered for dropout and their MSV curves were studied to obtain the graph in Figure 5. Based on this, we perform a second iteration and tune the resonators to 235.5 Hz, 198.77 Hz, 265.99 Hz, and 398.75 Hz. Finally, we perform a third iteration to complete the resonator tuning design, and the tuned frequencies are 196.03 Hz, 235.5 Hz, 265.99 Hz, and 196.03 Hz.

**Figure 6:**
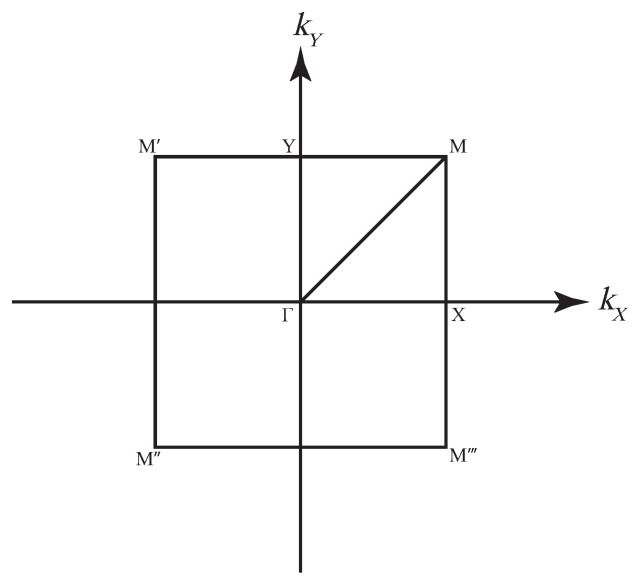
Illustration of the Brillouin zone.

**Figure 7:**
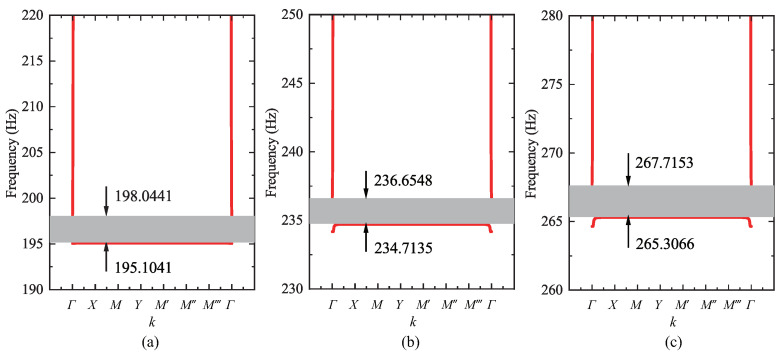
Bandgaps of resonators 1–3, respectively corresponding to (**a**–**c**).

The designed multimodal local resonators can generate an elastic wave band gap using a unique geometry design for vibration and noise reduction. The Bloch theory is applied to study the vibrational properties of phonon crystal formations by applying periodic boundary conditions, which can be applied to the unitary structure since phonon crystals are periodic. By introducing the Bloch wave vector *k* into the periodic boundary conditions and solving the eigenvalue problem, the intrinsic vibrational properties of the entire phononic crystal structure can be described. As the structure is asymmetric, its intrinsic vibrational properties can be analyzed in the Brillouin zone, as shown in Figure 6, by taking the wave vector *k* in various directions along the path. The energy band structure of a phononic crystal is the arrangement of intrinsic frequencies in different directions. The band structure calculation is performed using the finite element COMSOL Multiphysics 6.0, which allows us to find the vibrational modes of each band and facilitates theoretical analysis. The resulting energy band structure is is shown in Figure 7, and it can be observed that the oscillator produces a low-frequency band gap with a width of 3–6 Hz in the low-frequency range. The vibration is transmitted in a finite structure, and the MSV decays in the frequency band roughly corresponding to the energy band structure.

**Figure 8:**
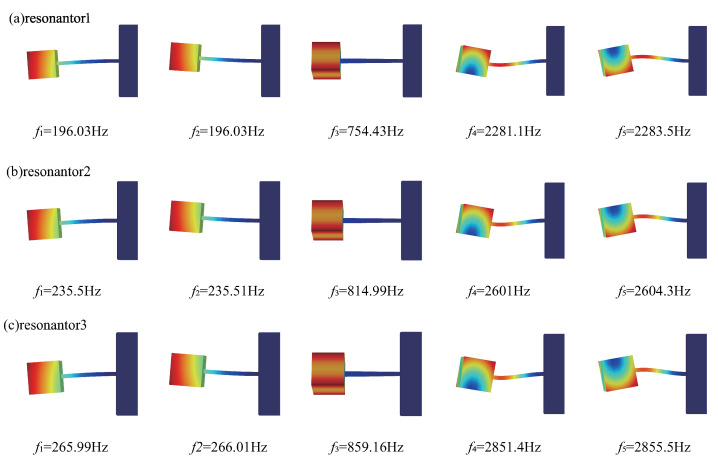
The first five modes of resonators 1–3.

Once the structural parameters of the multimodal local resonators were determined, we used COMSOL 6.0 to simulate and observe the first five orders of the resonator modes separately and calculate their modal frequencies; the results are shown in Figure 8.

### 2.3. MMABH Plate

**Figure 9:**
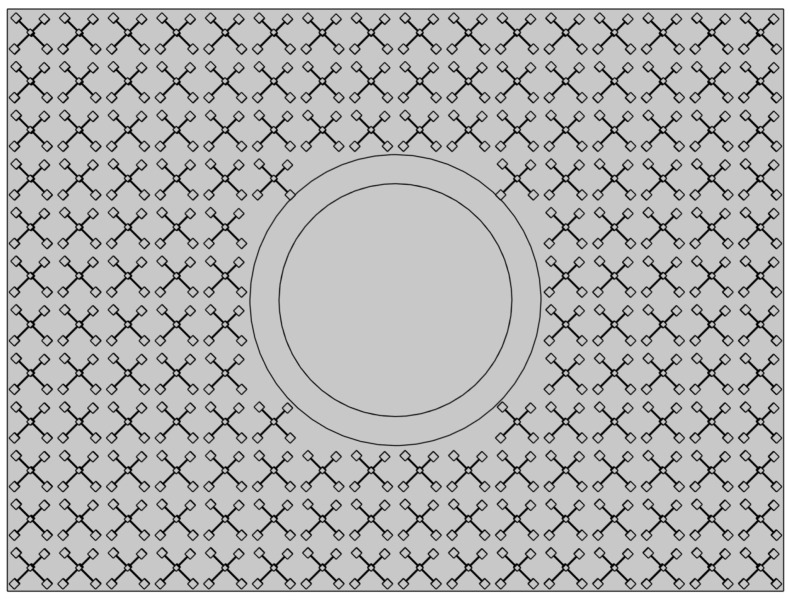
Assembled structure of the MMABH plate.

**Figure 10:**
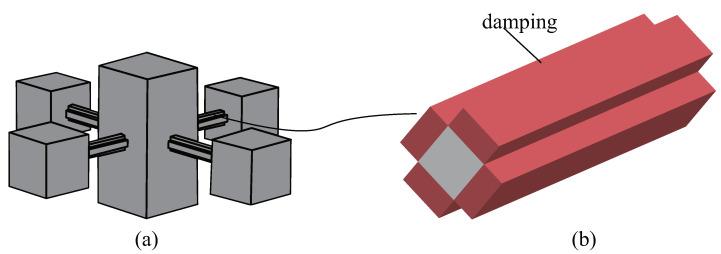
(**a**) Multimodal local resonators with damping. (**b**) A supporting bar with damping layers.

In this study, we assembled the multimodal local resonators with a double-layer ABH plate to form an MMABH plate (see Figure 9). The resonators are arranged periodically on the surface of the constant-thickness part of the plate using a 16 × 12 array of local resonance units, with each unit containing three oscillator modes for a total of 640 oscillators. The resonator’s elastic beam was overlaid with damping (red part), as shown in Figure 10b, to enhance its performance.

Previous studies have extensively investigated ABHs [2,23,46,47]. In particular, the damping layer strongly influences the local modalities in the high-frequency range, resulting in an increased mass and stiffness. To improve the performance of the MMABH plate, we followed the concept proposed in [43], which adds regularly dispersed damped resonators to an ABH plate tuned at its first eigenfrequency.

In the next phase of our work, we will compare the vibration levels of the MMABH plate with those of other models using COMSOL Multiphysics 6.0. The goal is to determine whether the developed MMABH plate can achieve significant vibration reductions in the low- to high-frequency range, thus demonstrating its potential for noise and vibration control applications.

## 3. Performance of the MMABH

### 3.1. MMABH Modal Analysis

In this section, we focus on a comparative analysis of the modal frequency, modal damping, and modal shape of the double-layer ABH plate plus resonators (MMABH).

**Figure 11:**
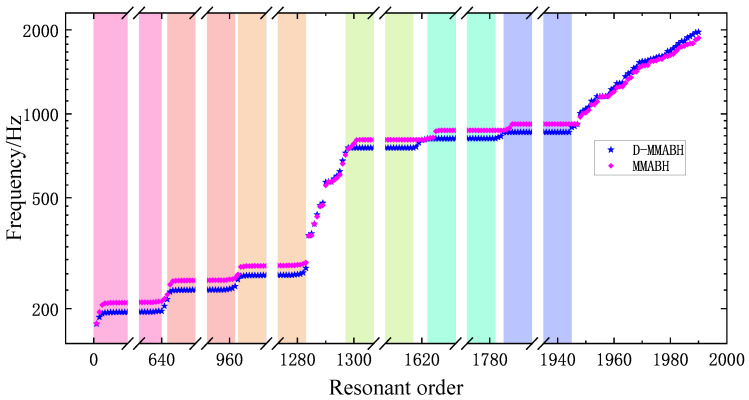
The modal frequency of the MMABH plate.

Firstly, we simulated the modal frequencies of the MMABH (see Figure 11). As shown in Figure 8, the symmetry of the resonator’s design structure causes the mass block to bend upwards and downwards together with the beam, resulting in almost equal modal frequencies. The same is true for bending towards the sides. However, the third mode is different, where the mass block is twisted and the beam does not vibrate. As a result, the vibration wave from the supporting bar cannot be transmitted to the mass block through the beam, making it impossible to excite. The vibration wave from the supporting bar can hardly cause the resonance of the mass block; therefore, it can not be excited. The characteristic frequencies of the MMABH plate, therefore, form a stepped image at different frequencies in groups of 320 or 640.

**Figure 12:**
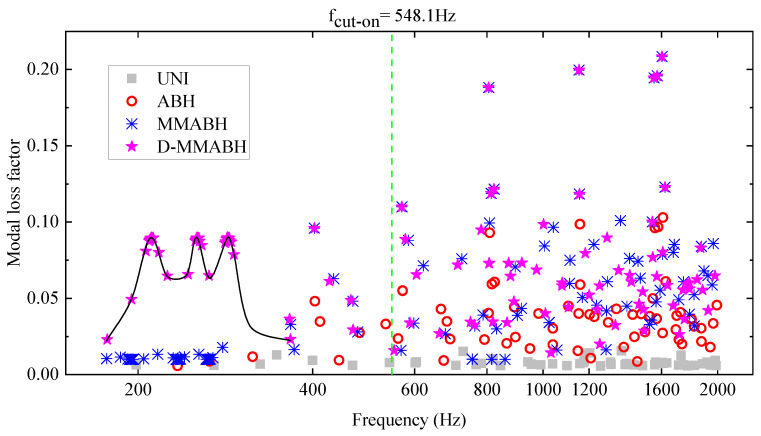
Modal loss factor of UNI, ABH, MMABH, D-MMABH plates.

Secondly, Figure 12 shows the modal loss factors (MLFs) for four different plates: uniform plate (UNI), ABH plate (ABH), MMABH plate (MMABH), and MMABH with damping (D-MMABH). The MLF of the UNI plate is almost constant and equal to 0.008 for all modes. However, due to the ABH effect, there are higher MLFs at high frequencies for the ABH plate, as observed for *f* > 548 Hz. Nevertheless, the MLFs of the ABH plate are very small in the low-frequency band. The MMABH solves this problem by adding multimodal resonators to the double-layer ABH plate, resulting in a very low amplitude and slight vibration energy loss at these frequencies. When overlaid damping is applied, the MLFs are significantly increased. As shown in Figure 12, the MLFs of the D-MMABH show interesting variations below 400 Hz, with three peaks at 196 Hz, 235 Hz, and 265 Hz. These peaks are explained by the fact that the resonator is tuned at these frequency points, and the resonator has a large MLF due to overlay damping. At other locations, the MLF of these resonators is smaller because the plate is vibrating rather than the damped resonator. The MLFs of the MMABH and D-MMABH show a repetition at these three frequency points, and the addition of damping has little effect on the frequency points outside the three frequency settings of the resonator. The situation is different in the high-frequency band, where the ABH region is vibrating and absorbing energy rather than the resonator.

**Figure 13:**
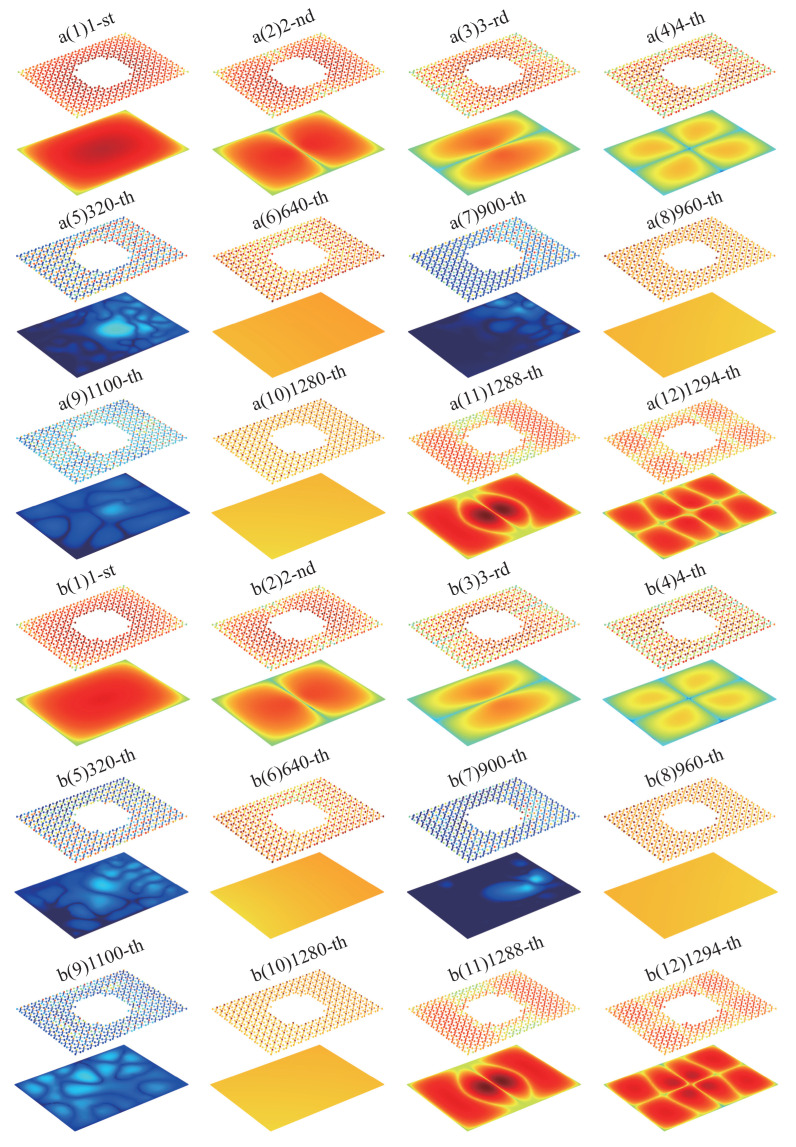
D-MMABH (**a**(1)–**a**(12)) and MMABH (**b**(1)–**b**(12)) mode shapes. The darker the color, the larger the amplitude.

Finally, taking the mode shape diagram of D-MMABH in Group a as an example, for the first order (see Figure 13a(1)), we can observe that the ABH plate vibrates in phase with the resonators with similar amplitude. As the modal order increases, the resonator’s vibration amplitude increases, while the whole plate decreases. At the 320th order, the resonator is vibrating most strongly (see Figure 13a(5)). As the order continues to increase, the vibration of the plate is strong near the 640th order, weak near the 900th order, strong again at the 960th order, weak again at the 1100th order, strong again at the 1280th order, and weak again after the 1288th order. This phenomenon vividly demonstrates the change in D-MMABH mode damping at low frequencies in the above figure. The damping is applied to the resonator, so the vibration in the plate loses almost no energy, resulting in a high modal.

### 3.2. Analysis of Wide-Band Vibration Reductions in MMABH Plate under External Force

**Figure 14:**
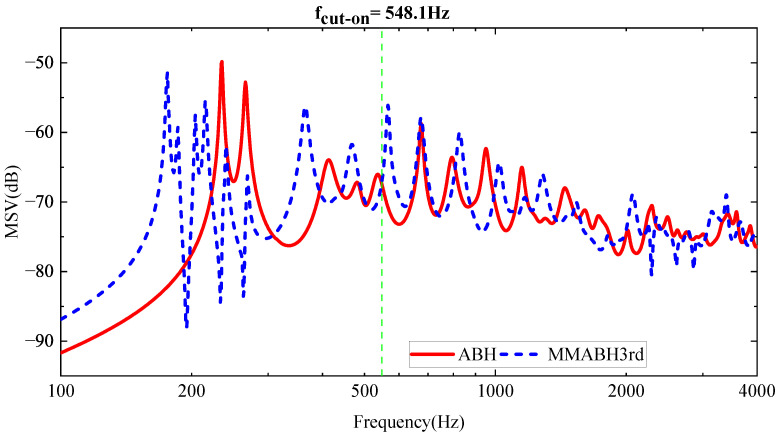
Mean square velocity (MSV) comparison between the ABH plate and the MMABH plate.

After being excited by an external point force, we can focus on the response of the MMABH plate. The force is assumed to have a constant amplitude of 2 N for all frequencies and is directed vertically towards the plate at (0.3, 0, 0.0275) m. The MSV figure of ABH-MMABH (see Figure 14) can be drawn to show the reduction in vibration caused by the addition of resonators in the three iterations to the double-layer ABH plate. As expected, the MMABH plate significantly reduces the MSV at the three frequency points where the arrows point, indicating the presence of band gaps. It is reasonable to expect that the vibration reduction will expand to lower frequencies.

**Figure 15:**
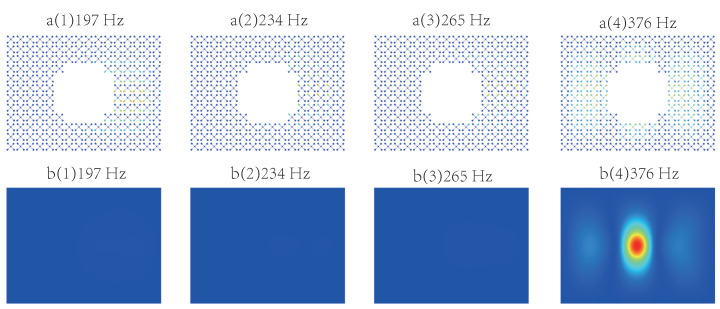
Forced vibration shapes of the MMABH plate at different frequencies.

To gain further insights into the behaviour of the MMABH plate, we calculated and plotted some of the forced vibration shapes in Figure 15. The first row shows the response of the resonators, while the second row contains the forced vibration shapes of the MMABH plate. At the band gap frequencies of 197 Hz, 234 Hz, and 265 Hz, the plates remain still because the resonators absorb all the vibration energy. At a frequency of 376 Hz, the plate part recovers its proper vibration amplitude since the resonators do not vibrate violently. However, the connecting rod vibrates with the plate, reducing the efficiency of some resonators. Therefore, the MSV of the plate at 373 Hz is much larger than that of the other three frequencies, as shown in Figure 14.

Regarding the activation of resonators, the two resonators below the connecting rod are activated at a vibration frequency of 197 Hz, the resonator in the upper left corner is activated at a vibration frequency of 234 Hz, and the resonator in the upper right corner is activated at a frequency of 265 Hz. The slight displacement of the connecting rod at 376 Hz is also noteworthy. These results indicate that the resonators in the MMABH plate behave as expected.

### 3.3. Parametric Study

#### 3.3.1. Effects of Multimodal Local Resonators

We now investigate the coupling action of the multimodal local resonators designed with two layers of ABH plates. However, it is worth noting that the MSV curve of the MMABH exhibits two troughs in the high-frequency region when the excitation frequency exceeds 548.1 Hz (see Figure 14). To explore the reason for this, we examined the first five orders of the resonator’s modes and their modal frequencies, as shown in Figure 8. The fourth and fifth modes of the resonator are at work, and these two modes are excited to absorb the vibration of the MMABH plate. In contrast, the third mode of the resonator does not play a similar role because the elastic beam does not vibrate. The third mode causes the mass block to twist, and the vibration wave from the supporting bar cannot be transmitted to the mass block through the elastic beam.

**Figure 16:**
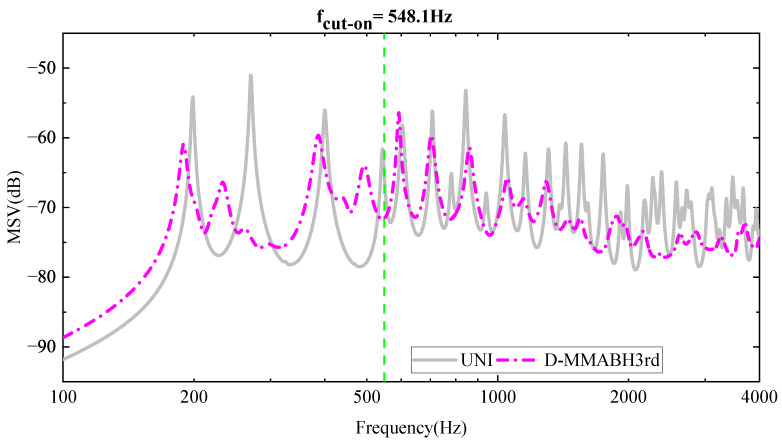
MSV curves for ABH and D-MMABH plates.

To further investigate the effects of the multimodal local resonators, we assembled MMABH plates with damped multimodal local resonators for observation. We provided MSV graphs at different frequencies for the two plates for analysis (see Figure 16). By comparing the MSV curves of the ABH plate and MMABH plate in the low-frequency band, we can see that the significant peak value of the ABH plate at 234 Hz is substantially reduced, and the overall vibration level of the board is much gentler. This is due to the combination of the resonator and damping. However, readers may require clarification regarding changes in the high-frequency part of the curve. This is because the addition of the resonator and damping increases the stiffness of the board, which shifts the frequency to lower frequencies. Moreover, the other modes of the resonator, although less significant than the first five modes, are equally effective. When excited, they also lead to a reduction in the vibration amplitude of the plate.

#### 3.3.2. Effects of the Damping of the Beam

**Figure 17:**
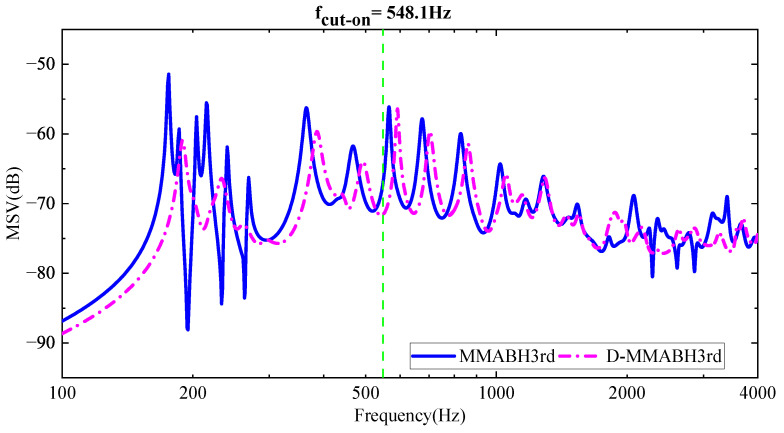
MMABH-DMMABH MSV Curve.

In the high-frequency range, the damping layer has a strong influence on the local modalities, leading to an increase in mass and stiffness [47]. Previous studies [27,47] have focused on vibration suppression in ABH plates above the cut-off frequency, but low-frequency vibrations are primarily related to the overall modal state of the plate. Therefore, it is necessary to investigate the effects of damping on the beam. Figure 17 illustrates the difference between a D-MMABH plate and an MMABH plate without damping. It can be observed from the figure that the damping resonator flattens the MMABH amplitude in the frequency band where the resonator operates. Damping absorbs the energy of the vibration wave, allowing for the resonator to return to rest more quickly. Without the damping material, the vibration energy is difficult to consume; in other words, the mass block will continue to vibrate for a long time, which will affect the subsequent damping effect. If damping occurs, the vibration will soon stop. The forced mode shapes of the MMABH and the DMMABH are shown at 197 Hz, 234 Hz, 265 Hz, and 376 Hz, respectively, in Figure 15. By comparing the graphs of the same frequencies of group a and group b (i.e., a1 and b1, a2 and b2), the vibration of the MMABH is reduced throughout the entire frequency range, even if the damping reduces the resonator’s absorption effect at its characteristic frequency.

## 4. Conclusions

In this paper, the design of an MMABH plate capable of suppressing bending vibrations on the broadband spectrum has been presented. It consists of a double-layer ABH plate plus resonators. The resonators are tuned to the three low-frequency peaks of the double-layer ABH plate to create band gaps, while the damping is superimposed to reduce vibration levels at other frequencies. MMABH plates combine the properties of the high-frequency ABH effect with the formation of low-frequency band gaps, which are a promising locally resonant acoustic metamaterial.

A modal analysis of the MMABH plate was first carried out to investigate its characteristics, including modal frequency, modal damping, and modal shape. The results show that the characteristic frequencies of the MMABH plate form a stepped image and modal loss factors of the ABH and MMABH plates were almost equal at high frequencies. The broadband damping performance of the MMABH plate under external forces was then investigated, and the results showed that the MSV of the MMABH plate and D-MMABH was lower than that of the uniform and conventional ABH plate.

The next task was to analyse the coupling effect between the multimodal local resonator and the ABH plate at different frequencies using the MSV curves and forced mode diagrams of the MMABH. The results show that the resonator is damped at low frequencies but not at high frequencies. In addition, the effect of the multimodal local resonator is further investigated in this paper by analysing the MSV curves of the ABH and MMABH plates under forced vibration. It is found that the resonator has a damping effect at low frequencies and that the damping of the beam flattens the amplitude of the MMABH in the frequency band in which the resonator operates.

Overall, the D-MMABH designed in this study exhibits good broadband-damping effects and load-bearing performance. It is a promising metamaterial for local resonance acoustics.

## Figures and Tables

**Table 1:** Geometrical and material parameters of the ABH plate. $\rho_{p}$: plate density, $\rho_{\nu }$: damping layer density, $E_{p}$: plate Young modulus, $E_{\nu }$: damping layer Young modulus, $\eta_{p}$: plate loss factor, $\eta_{\nu }$: damping loss factor, $\nu_{p}$: plate Poisson ratio, $\nu_{\nu }$: damping Poisson ratio.

Geometry Parameters	Material Parameters
m=2.5	$\rho_{p}=7800\;\mathrm{kg}/m^{3}$
$L_{x}=0.8\;m$	$E_{p}=210\times 10^{9}\;\mathrm{Pa}$
$L_{y}=0.6\;m$	$\eta_{p}=0.01$
$h_{\mathrm{uni}}=0.005\;m$	$\nu_{p}=0.3$
$r_{\mathrm{abh}}=0.15\;m$	
$\varepsilon =0.5451\;m^{-1.5}$	$\rho_{\nu }=950\;\mathrm{kg}/m^{3}$
$h_{c}=0.00025\;m$	$E_{\nu }=5\times 10^{9}\;\mathrm{Pa}$
$r_{v}=0.12\;m$	$\eta_{\nu }=0.5$
$h_{v}=0.0025\;m$	$\nu_{\nu }=0.3$
$a_{\mathrm{studdle}}=0.005\;m$	
$L_{\mathrm{studdle}}=0.02\;m$	

**Table 2:** Structure parameters and natural frequencies of two types of resonator.

Category	$L_{k}\left[\mathrm{mm}\right]$	$r_{k}\left[\mathrm{mm}\right]$	First Order Eigenfrequency [Hz]
resonator 1	17.4	1	196.03
resonator 2	14.9	1	235.5
resonator 3	13.4	1	265.99

## Data Availability

Data will be available on reasonable request.
